# Flexible and Interpretable Modeling of Overlapping Exposure Risks in Self‐Controlled Case Series Analysis

**DOI:** 10.1002/sim.70552

**Published:** 2026-04-14

**Authors:** Xuezhixing Zhang, Paul Milligan, Yin Bun Cheung

**Affiliations:** ^1^ Centre for Biomedical Data Science, Duke‐NUS Medical School National University of Singapore Singapore; ^2^ Faculty of Epidemiology and Population Health London School of Hygiene & Tropical Medicine London UK; ^3^ Tampere Center for Child, Adolescent and Maternal Health Research Tampere University Tampere Finland

**Keywords:** multiple exposures, self‐controlled case series, single index link function, splines

## Abstract

The self‐controlled case series (SCCS) method is frequently employed to explore the relationship between transient exposures and subsequent health events, utilizing data from individuals who have experienced the event of interest. Conventional spline‐based SCCS models typically do not account for overlapping exposure periods and fail to accommodate complex interactive effects among multiple exposures. In this paper, we introduce a novel semiparametric SCCS method that employs a functional partial‐linear single index (PLSI) link function, allowing for the estimation of overlapping exposure risks. Our approach offers greater interpretability and flexibility compared with existing methods by consolidating multiple exposures into a single index and modeling complex interactions through a nonparametric link function. We validate our model through simulation studies comparing its performance with standard methods under various practical exposure settings. Furthermore, we apply our method to two real‐world datasets involving MMR vaccination and malaria chemoprevention, demonstrating its practical utility and enhanced capability to handle multiple, overlapping exposures effectively. Our findings suggest that the PLSI‐SCCS model is a robust tool for modern epidemiological and pharmaceutical research, providing a nuanced understanding of exposure effects, particularly in complex multi‐exposure scenarios.

## 1 Introduction

The self‐controlled case series (SCCS) method is frequently employed to evaluate the relationship between transient exposures and subsequent health events, utilizing data from individuals who have experienced the event of interest at least once [1]. The method was first introduced in 1995 for the study of vaccine safety, and has since been applied to a wide range of epidemiological studies [2, 3]. In recent years, SCCS has also increasingly been used to assess treatment effectiveness [4, 5] and the impact of preventive interventions [6, 7, 8] when exposures vary over time and outcomes are acute events. A key feature of the SCCS design is its self‐controlled nature. By conditioning on the total number of events each individual experiences during their observation period, all time‐invariant confounders are inherently controlled. Time‐varying confounders, such as age and season, usually require appropriate adjustment in the analysis.

The use of SCCS methods relies on several key assumptions [2, 9]. First, events within individuals should follow a non‐homogeneous Poisson process. Second, the observation period must be independent of the event times. Third, by conditioning on the total number of events within each individual, SCCS inherently controls for baseline incidence and all time‐invariant confounders. Fourth, it is assumed that previous events do not affect the probability of subsequent exposures. Standard SCCS formulations consistently rely on these four core assumptions. In applied research, a common parametric specification of the standard SCCS also assumes constant risk levels within time‐periods defined by exposure status and time‐varying confounders, allowing for estimation using piecewise constant step functions. Although SCCS is most commonly applied to transient exposures with well‐defined risk windows, exposures may also have prolonged or effectively indefinite risk periods in some applications [2, 7, 10]. In such settings, or more generally when the exposure effect may vary over time, previous SCCS extensions have involved flexible modeling of time‐varying effects. Specifically, to avoid misspecification of exposure effects over time, Ghebremichael‐Weldeselassie et al. [11] employed M‐splines to approximate the exposure effect. Similar approaches were subsequently utilized for age effect estimation and extended to a series of spline‐based semiparametric or nonparametric SCCS models [12, 13]. Although some studies have discussed spline‐based SCCS methods with multiple exposures, all of them assume non‐overlapping periods. Few studies have addressed the spline‐based SCCS method for multiple exposures whose effects may overlap in time.

Accounting for multiple exposures is important in infectious disease prevention studies. Such scenarios may include repeated administrations of the same treatment or the use of multiple treatments per person. In pharmaceutical research, the delivery of biopharmaceutical products often involves administering multiple doses at scheduled times. For instance, some prophylactic drugs, such as malaria chemoprevention and antibiotic preventive treatments, have fairly short‐lived effects and should be repeatedly administered to maintain protective efficacy [14]. Moreover, in pediatric populations, administration of multiple vaccines, such as measles, mumps, and rubella (MMR) and varicella vaccines, may take place on the same day or within a short period [15, 16]. Such scenarios can result in complex nonspecific and interactive effects among multiple exposures, increasing the complexity of assessing the safety and efficacy of vaccines and drugs [17, 18].

Existing SCCS methods for multiple exposure problems face a significant dilemma. The standard SCCS method models overlapping risk periods by including linear interaction terms. A recent extension [19] proposed a three‐part SCCS model that partitions risk periods into prespecified intervals for each exposure and an explicit overlap period, enabling estimation of overlap effects under both multiplicative and additive interaction assumptions while maintaining a piecewise constant risk structure. This method is intuitive and interpretable, but the constant risk assumption may be restrictive when exposure effects vary over time. While in current spline‐based methods, incorporating interaction terms with splines can substantially increase model complexity, leading to overfitting and a loss of efficiency, these interaction terms are often difficult to visualize and interpret [20]. As a result, existing spline‐based models for multiple exposures frequently rely on a strong assumption that multiple exposure risk periods do not overlap [13], thereby excluding interaction terms from the model.

To address the aforementioned challenges, we introduce the functional partial‐linear single index (PLSI) link function to estimate overlapping risk periods within the SCCS framework. The PLSI model was first proposed by Wang et al. 2010 [21] to mitigate the ‘curse’ of dimensionality in multivariate models. This approach later gained popularity in econometrics and environmental epidemiology and was subsequently extended to a functional form by Wang et al. 2016 [22]. Compared with existing SCCS methods, the PLSI function offers greater interpretability and parsimony in the analysis of multiple exposures. Specifically, the proposed model consolidates multiple exposures into a single index, allowing the associations between exposures and outcomes to vary directionally. It provides explicit and interpretable quantification of the relative directions and importance of each exposure while flexibly modeling these complex interactive effects through the nonparametric PLSI function [21, 22, 23].

The rest of this paper is organized as follows. In Section 2, we define the PLSI‐SCCS likelihood function and describe the estimation procedure. Section 3 presents simulations to evaluate the performance of the proposed model under practical settings, where we first compare PLSI‐SCCS models with current semiparametric methods for single exposure estimation and then assess our methods under multiple exposure settings. In Section 4, we illustrate the usefulness of the method by analyzing two real datasets, one on febrile convulsions in relation to MMR and *Haemophilus influenzae* type B (Hib) vaccines, and the other on clinical malaria in relation to repeated doses of malaria chemoprevention. We conclude with final remarks and discuss future directions in Section 5.

## 2 Methodology

### 2.1 The Standard SCCS Likelihood

In this paper, whenever we refer to ‘standard SCCS’, we specifically mean the parametric step‐function implementation with prespecified risk intervals, as most commonly used in applied work and implemented in the R ‘SCCS’ package [24]. Let $\left(a_{i},b_{i}\right]$ denote the observation period for individual i=1,2,…,N. Define $x_{i}\left(t\right)$ as the exposure vector that individual i experiences at time t within the observation period, and let $z_{i}\left(t\right)$ be the vector of time‐varying confounders (e.g., age groups). The unconditional cohort likelihood for individual i, who recurrently experiences $n_{i}$ events arising from an intensity process $\lambda_{i}\left(t|x_{i}\left(t\right),z_{i}\left(t\right)\right)$ at event times $t_{\mathrm{ij}}\;\left(j=1,\ldots ,n_{i}\right)$ can be written as

$$L_{i}^{u}=\prod_{j=1}^{n_{j}}\lambda_{i}\left(t_{\mathrm{ij}}|x_{i}\left(t\right),z_{i}\left(t\right)\right)\exp\left[-\int_{a_{i}}^{b_{i}}\lambda_{i}(t_{\mathrm{ij}}|x_{i}\left(t\right),z_{i}\left(t\right))\mathrm{dt}\right].$$

Under the proportional incidence model, we assume:

$$\lambda_{i}\left(t_{\mathrm{ij}}|x_{i}\left(t\right),z_{i}\left(t\right)\right)=\lambda_{0}\exp\left(\gamma_{i}+x_{i}{\left(t\right)}^{T}\beta +z_{i}{\left(t\right)}^{T}\theta \right),$$

where $\lambda_{0}$ is the baseline incidence, $\gamma_{i}$ captures all time‐invariant individual‐specific effects, and β and θ are coefficient vectors for the log relative incidence (log‐RI) of exposures of interest and confounders, respectively.

The standard SCCS likelihood is derived by conditioning on the total number of events $n_{i}$ ^2^. Consequently, the constant terms $\lambda_{0}$ and $\exp\left(\gamma_{i}\right)$ cancel out, yielding the conditional likelihood:

(1) $$\begin{array}{ll} L^{c} & =\prod_{i=1}^{N}L_{i}^{c}\\ & =\prod_{i=1}^{N}\prod_{i=1}^{n_{i}}\frac{\lambda_{i}\left(t_{\mathrm{ij}}|x_{i}\left(t\right)\right)}{\int_{a_{i}}^{b_{i}}\lambda_{i}\left(t|x_{i}\left(t\right)\right)\mathrm{dt}}\\ & =\prod_{i=1}^{N}\prod_{i=1}^{n_{i}}\frac{\exp\left(x_{i}{\left(t_{\mathrm{ij}}\right)}^{T}\beta +z_{i}{\left(t\right)}^{T}\theta \right)}{\int_{a_{i}}^{b_{i}}\exp\left(x_{i}{\left(t\right)}^{T}\beta +z_{i}{\left(t\right)}^{T}\theta \right)\mathrm{dt}}. \end{array}$$

In the standard form of the SCCS likelihood function, both $x_{i}\left(t\right)$ and $z_{i}\left(t\right)$ are represented by step functions. In this formulation, the numerator is the product of the relative incidences at the observed event times, while the denominator is the product of the integrated relative incidences across the entire observation period for each individual. This model can also be applied to analyze non‐recurrent event data ($n_{i}=1$ for i=1,…,N), when the event risk is small over the observation period^2^.

### 2.2 Spline‐Based SCCS Methods

Previous semiparametric and nonparametric extensions of the standard SCCS [11, 12, 13] leave the RI functions for time‐varying confounders and exposures unspecified and approximate them with linear combinations of spline functions. The general form of the spline‐based SCCS likelihood with single exposure can be written as

(2) $$L^{c}=\prod_{i=1}^{N}\prod_{i=1}^{n_{i}}\frac{\psi \left(t_{\mathrm{ij}}\right)\omega \left(t_{\mathrm{ij}}-c_{i}\right)}{\int_{a_{i}}^{b_{i}}\psi \left(t\right)\omega \left(t-c_{i}\right)\mathrm{dt}}$$

where ψ(·) is the smoothed RI function related to time‐varying confounders and ω(·) denotes the exposure‐related RI function. $c_{i}$ is the start time of the risk period for individual i, and $t-c_{i}$ represents the relative time within that risk period. Both ψ(·) and ω(·) can be approximated by spline basis functions, allowing flexible functional forms in data analysis.

### 2.3 The PLSI‐SCCS Model for Multiple Exposures

When multiple exposures are present, modeling the exposure‐related RI function becomes more complex. Let p be the number of exposures, and denote $\left(c_{\mathrm{ik}},d_{\mathrm{ik}}\right]$ as the risk period of the *k*th exposure of individual i. When the length of the kth risk period is indefinite or long‐lasting (i.e., $d_{\mathrm{ik}}=\infty$), $d_{\mathrm{ik}}$ can be set equal to the end time of the observation period $b_{i}$. If these risk periods do not overlap, the exposure‐related RI for individual i at time t can be written as

$$\omega_{i}\left(t\right)=\left\{\begin{array}{l} \omega_{k}\left(t-c_{\mathrm{ik}}\right)\;\text{if}\;t\in \left(c_{\mathrm{ik}},d_{\mathrm{ik}}\right],k=1,\ldots ,p,\\ 1\;\text{otherwise}. \end{array}\right.$$

However, when risk periods overlap, the interactive effects may be non‐additive and more intricate. To estimate interactive effects, we incorporate the PLSI link function into the SCCS model. We define as follows:

$$\omega_{i}\left(t\right)=S_{1}\left(\sum_{k=1}^{p}S_{2k}\left(t-c_{\mathrm{ik}}\right)\right),$$

where $S_{1}\left(\cdot \right)$ is a nonparametric single‐index link function, and $S_{2k}\left(\cdot \right)$ characterizes the relative direction and effect of the kth exposure [22]. If $S_{1}\left(\cdot \right)$ is monotonically increasing, larger values of $S_{2k}\left(\cdot \right)$ will correspond to higher exposure‐related RI when all other variables are fixed. Setting $S_{2k}\left(t\right)=0$ implies no effect for exposure k at time t. When $S_{1}\left(\cdot \right)$ is not monotone, the $S_{2k}\left(\cdot \right)$ would only represent the relative importance of exposure k in the overall exposure effect. Because $S_{1}\left(\cdot \right)$ is nonparametrically modeled, it naturally captures any nonlinear interaction effects among overlapping risk periods.

To ensure sufficient flexibility while preserving nonnegativity of the RI function, we approximate $S_{1}\left(u\right)$ using B‐splines with squared coefficients:

$$S_{1}\left(u\right)\approx B_{1}^{T}\left(u\right)\beta^{2}=\sum_{l=1}^{M_{1}}\beta_{l}^{2}B_{1l}^{T}\left(u\right)$$

where $B_{1}\left(u\right)={\left(B_{11}\left(u\right),\ldots ,B_{1M_{1}}\left(u\right)\right)}^{T}$ is a vector of $M_{1}$ basis functions (i.e., total degrees of freedom), and $\beta^{2}={\left(\beta_{1}^{2},\ldots ,\beta_{M_{1}}^{2}\right)}^{T}$. In the control (unexposed) period, we set the exposure‐related RI to 1.

Similarly, each $S_{2k}\left(t-c\right)$ can be approximated by normalized B‐splines:

$$S_{2k}\left(t-c_{\mathrm{ik}}\right)=\left\{\begin{array}{l} B_{2k}^{T}\left(t-c_{k}\right)\gamma_{k}\;\text{if}\;c_{\mathrm{ik}}\leq t\leq d_{\mathrm{ik}},\\ 0,\;\text{otherwise}. \end{array}\right.$$

where $B_{2k}$ is a vector of $M_{2k}$ spline basis functions, and $\gamma_{k}$ is the corresponding coefficient vector.

By substituting these spline approximations into the SCCS framework, we obtain the conditional likelihood for the PLSI‐SCCS model:

(3) $$\begin{array}{cc} L^{c} & =\prod_{i=1}^{N}\prod_{i=1}^{n_{i}}\frac{\psi \left(t_{\mathrm{ij}}\right)\left[S_{1}\left(\sum_{k=1}^{p}S_{2k}\left(t_{\mathrm{ij}}-c_{\mathrm{ik}}\right)\right)\right]}{\int_{a_{i}}^{b_{i}}\psi \left(t\right)\left[S_{1}\left(\sum_{k=1}^{p}S_{2k}\left(t-c_{\mathrm{ik}}\right)\right)\right]\mathrm{dt}}\\ & =\prod_{i=1}^{N}\prod_{i=1}^{n_{i}}\frac{\psi \left(t_{\mathrm{ij}}\right)\left\{B_{1}^{T}\left[{\left(B_{2}R_{i}\left(t_{\mathrm{ij}}\right)\right)}^{T}\gamma \right]\beta^{2}\right\}}{\int_{a_{i}}^{b_{i}}\psi \left(t\right)\left\{B_{1}^{T}\left[{\left(B_{2}R_{i}\left(t\right)\right)}^{T}\gamma \right]\beta^{2}\right\}\mathrm{dt}}. \end{array}$$

where $B_{2}$ is a block‐diagonal matrix of dimension $\left(\sum_{k=1}^{p}M_{2k}\right)\times p$, whose kth block contains the basis functions for the kth exposure. The vector $R_{i}\left(t\right)$ indicates which exposures are effective at time t for individual i. That is, $R_{\mathrm{ik}}\left(t\right)=1$ if the individual i is in the effective period of the kth exposure, and $R_{\mathrm{ik}}\left(t\right)=0$ otherwise. All spline coefficients are collected in $\gamma =\left(\gamma_{1},\ldots ,\gamma_{p}\right)$. In addition to the nonnegativity of the single‐index link function $S_{1}\left(\cdot \right)$, which is ensured by representing spline coefficients as squared parameters $\beta^{2}$, we impose the following identifiability constraints [23, 25]: (i) Unit norm constraint on γ: we constrain the $l_{2}$ norm of γ to be 1 (i.e., ${\left\|\gamma \right\|}^{2}=\sqrt{\gamma_{11}^{2}+\ldots +\gamma_{{\mathrm{pM}}_{2p}}^{2}}=1$); (ii) direction constraint on γ: we requrie the first component of γ to be positive, (i.e., $\gamma_{11}>0$). Together, these constraints ensure identifiability of the PLSI‐SCCS method by removing scale ambiguities inherent in PLSI models. We briefly discuss the consequences of violating these constraints in the discussion section, and provide further details with a summary table in Appendix 1.

### 2.4 Estimation

For convenience of illustration of the estimation procedure, we focus on PLSI‐SCCS models where the effects of time‐varying confounders, ψ(t), is approximated by step functions. However, our method can be readily extended to more flexible form in which ψ(t) is approximated by a linear combination of spline functions. The corresponding log‐likelihood function is then

$$\begin{array}{cc} l^{c}\left(\gamma ,\beta ,\theta \right) & =\sum_{i=1}^{N}\sum_{j=1}^{n_{i}}\left\{\log\left[\exp\left(z_{i}{\left(t_{\mathrm{ij}}\right)}^{T}\theta \right)\right]+\log\left\{B_{1}^{T}{\left[\left(B_{2}R_{i}\left(t_{\mathrm{ij}}\right)\right)\right]}^{T}\gamma \right\}\beta^{2}\right\}\\ & \;-\sum_{i=1}^{N}n_{i}\log\left\{\int_{a_{i}}^{b_{i}}\left[\exp\left(z_{i}{\left(t\right)}^{T}\theta \right)\right]\left\{B_{1}^{T}\left[{\left(B_{2}R_{i}\left(t\right)\right)}^{T}\gamma \right]\beta^{2}\right\}\mathrm{dt}\right\} \end{array}$$

To avoid overfitting of the single‐index link function, we further propose a penalized PLSI‐SCCS approach [26, 27]. Specifically, we include a penalty term based on the second derivative of $B_{1}\left(\cdot \right)$ to regulate the smoothness of the single‐index link function. The penalized objective function is

$$Q\left(\gamma ,\beta ,\theta \right)=l^{c}\left(\gamma ,\beta ,\theta \right)-\lambda \left({\left(\beta^{2}\right)}^{T}D\beta^{2}\right)$$

where D is an $M_{1}\times M_{1}$ positive semidefinite symmetric matrix whose (m,n)‐th element is $\int_{\min\left\{{\left(B_{2}R_{i}\left(t\right)\right)}^{T}\gamma \right\}}^{\max\left\{{\left(B_{2}R_{i}\left(t\right)\right)}^{T}\gamma \right\}}\;B_{1m}^{\prime \prime }\left(u\right)B_{1n}^{\prime \prime }\left(u\right)\mathrm{du}$. This is the usual quadratic integral penalty in Ruppert, 2002 [28]. Here, λ is the tuning parameter that controls the degree of smoothing, with larger λ enforcing smoother estimates of the link function. As noted in Joly et al. 1998 [29] and Rondeau et al. 2005 [30], choosing $M_{1}$ between 8 and 15 is usually sufficient to offer enough flexibility, while the smoothness of the fitted curve is jointly adjusted by $M_{1}$ and λ.

Following the estimation procedure in other PLSI models [31, 32], we use a profiling method to minimize the objective function Q(·). Define $\eta ={\left(\beta ,\theta \right)}^{T}$ and treat η as a function of γ, denoted as $\overset{`}{\eta }\left(\gamma \right)$. We obtain the proposed estimators by iterating the following two steps:

1. Update $\overset{`}{\eta }$: Given $\overset{`}{\gamma }$, solve $\overset{`}{\eta }=\arg\min_{\eta }\;Q\left(\eta \right)$. We enforce the constraint on $\overset{`}{\gamma }$ by multiplying γ by the sign of its first component $\gamma_{11}$ and then dividing by its $l_{2}$ norm.
2. Update $\overset{`}{\gamma }$: Given $\overset{`}{\eta }\left(\gamma \right)$, find the $\overset{`}{\gamma }$ that minimizes $Q\left(\gamma ,\overset{`}{\eta }\left(\gamma \right)\right)$.

These optimization steps are carried out using the ‘optim’ function in R, employing the Broy‐den‐Fletcher‐Goldfarb‐Shanno (BFGS) algorithm [33]. Numerical integration in the objective function is approximated using the trapezoidal rule [34]. When fitting the model, we employ cubic B splines to approximate the single‐index link function $S_{1}\left(\cdot \right)$, along with natural cubic B splines for the $S_{2}$ curves. For $S_{1}\left(\cdot \right)$, the boundary knots are set to the minimum and maximum values drawn from all possible combinations of $S_{2}$ curves. The interior knots are determined by the quantiles of the observed $\sum_{k=1}^{p}\;S_{2k}$ in the dataset. For $S_{2}$ curves, the interior knots are selected according to the quantiles of the distribution of relative times for events within the risk period. In this paper, we assume the same number of basis functions for all $S_{2}$ curves and propose using mean prediction error as the cross‐validation metric to select the best $M_{2}$ and the smoothing parameter λ, given a fixed $M_{1}=10$.

In terms of inference, it is straightforward to derive the covariance of $\overset{`}{\theta }$ (Appendix 2), but the inference for $\overset{`}{\gamma }$ and $\overset{`}{\beta }$ requires difficult estimation of the conditional density of multiple integrations over the single‐index component, making it considerably more challenging. Consequently, we propose to adopt the bootstrapping method to construct confidence intervals (CIs) for predictions of the exposure‐related RI, where the CIs are defined as the 2.5th and 97.5th percentiles of the bootstrap distribution. For step‐function coefficients $\overset{`}{\theta }$, we continue to use the analytic confidence intervals derived in Appendix 2.

## 3 Simulation Study

### 3.1 Simulation Setting

**FIGURE 1:**
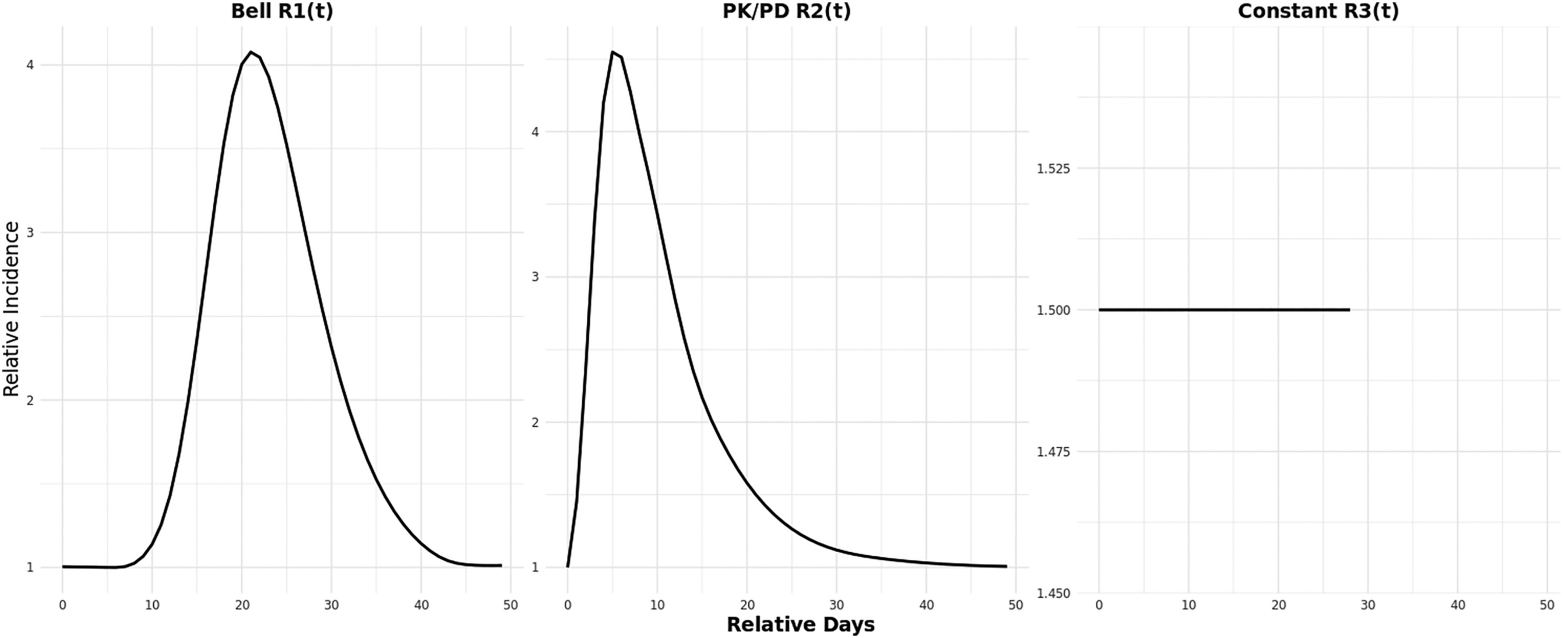
True exposure‐related relative incidence (RI) in simulation study.

In this section, we conducted a series of simulation studies with n=1000 persons each with one event to evaluate the performance of our model (3). All generated cases were assigned to have at least one risk period. Specifically, we considered three exposure‐related RI functions (Figure 1): a bell‐shaped risk curve $R_{1}\left(t\right)$ of 50 days; a 50‐day nonlinear, non‐monotonic and asymmetric risk curve $R_{2}\left(t\right)$ that represents the concentration‐time curve from a PK/PD model involving a sigmoid Emax pharmacodynamic (PD) model and a first‐order absorption pharmacokinetic (PK) model, which is suitable for studies of products administered via extravascular routes, such as most vaccines and oral drugs [14]. The last RI function is a constant risk period $R_{3}\left(t\right)=1.5$ lasting 29 days. The observation period for all individuals was set from 0 to 365 days. To simulate time‐varying confounding, we divided the timeline into four age groups, defined by cut points at 91, 182, and 274 days, with θ=(0,0.4,0.8,1.2).

We generated event data under six different settings. In Settings I and II, we simulated a single exposure period using $R_{1}\left(t\right)$ and $R_{2}\left(t\right)$, respectively. The start time of the risk period was uniformly randomly drawn from the observation period. Settings III through V considered multiple exposures with different RI functions. These settings were primarily designed to focus on the PK/PD curve $R_{2}\left(t\right)$ and its interactions with other risk periods, given that the PK/PD trajectory is a more biologically plausible shape and is broadly recognized in pharmaceutical research. In Setting III, there are two non‐overlapping periods $R_{1}\left(t\right)$ and $R_{2}\left(t\right)$, with $R_{2}\left(t\right)$ beginning 56 days after $R_{1}\left(t\right)$ starts. From Setting IV to Setting VI, we generated cases with overlapping risk periods. For all overlapping risk periods, the start time of the second risk period is uniformly drawn from a range of days. Specifically, Setting IV includes two overlapping periods, $R_{2}\left(t\right)$ and $R_{3}\left(t\right)$, where $R_{3}\left(t\right)$ begins 21–56 days after $R_{2}\left(t\right)$ starts. For the overlapping period, we assume a multiplicative relationship $R\left(t\right)=R_{1}\left(t\right)R_{2}\left(t\right)$. In Setting V, two risk periods $R_{1}\left(t\right)$ and $R_{2}\left(t\right)$ are used to generate data, and $R_{2}\left(t\right)$ begins 21–56 days after $R_{1}\left(t\right)$ starts. The overlapping period follows an additive relationship $R\left(t\right)=R_{1}\left(t\right)+R_{2}\left(t\right)-1$. Finally, Setting VI considers multiple exposures with the same RI function $R_{2}\left(t\right)$, which imitates a common scenario in studies with repeated doses. The second risk period starts 14–28 days after the first one, again with an additive relationship $R\left(t\right)=R_{1}\left(t\right)+R_{2}\left(t\right)-1$, but the maximum R(t) is capped at 4.5. We generated event times conditional on the exposure status from a multinomial distribution with daily categories. For each setting, 200 replicate datasets are simulated. In real data analyses, the true length of risk periods is often unknown when fitting the model, leading researchers to often select a risk period that is sufficiently long to encompass the true risk period. In consideration of this, we set the length of the risk period to be 7 days longer than that defined in the data generating process.

To assess model performance, we focus on how accurately it recovers the exposure‐related RI (including overlapping periods) and estimates the age effects θ. For each replicate, we compute the integrated squared error (ISE) for the *k*th exposure:

$${\mathrm{ISE}}_{k}=\sqrt{\int_{c}^{d}\;\;{\left[S_{1}\left(S_{2k}\left(t-c\right)\right)-{\overset{`}{S}}_{1}\left({\overset{`}{S}}_{2k}\left(t-c\right)\right)\right]}^{2}\mathrm{dt}}$$

Here c and d denote the start time of the first and end time of the last exposure period, respectively. Across 200 replicates, we report the median of these ISE values, denoted as the median integrated squared error (MISE). We also measure the mean absolute predicted error (MAPE) for the overall exposure‐related RI. Specifically,

$$\begin{array}{rr} \text{MAPE} & =\frac{1}{d-c}\int_{c}^{d}\;\;\left|S_{1}\left(\sum_{k=1}^{p}\;\;S_{2k}\left(t-c_{k}\right)\right)-{\overset{`}{S}}_{1}\left(\sum_{k=1}^{p}\;\;{\overset{`}{S}}_{2k}\left(t-c_{k}\right)\right)\right|\mathrm{dt}\\ & \approx \frac{1}{d-c}\sum_{t}\;\;\left|S_{1}\left(\sum_{k=1}^{p}\;\;S_{2k}\left(t-c_{k}\right)\right)-{\overset{`}{S}}_{1}\left(\sum_{k=1}^{p}\;\;{\overset{`}{S}}_{2k}\left(t-c_{k}\right)\right)\right|. \end{array}$$

The integral is approximated by a discrete sum overall risk periods. For age effects, we report the mean and standard deviation of the estimates $\overset{`}{\theta }$, as well as coverage probabilities (CPs) across the 200 replicates. For single‐exposure settings (I and II), we further compare the performance of our proposed model with the spline‐based model (3), which incorporates step functions for age effects and M‐splines for the key exposure effects. To assess finite‐sample stability in sparse‐event settings (as in rare vaccine adverse events), we additionally repeat Settings I and II with *n* = 200 and 500 cases. For multiple‐exposure settings (III to VI), we compare our model to the standard SCCS model that includes interaction terms. Details for simulation settings can be found in Appendix 3. Codes for model fitting and simulation can be found on Github (Xuezhixing‐Zhang/PLSI‐SCCS). The conventional spline‐based model was fitted with the R package ‘SCCS’ [24].

### 3.2 Simulation Results

Table 1 presents simulation results for single exposure settings I and II. In terms of the estimation of exposure‐related RI and age effects (θs), our method performs similarly to the conventional spline‐based SCCS method under setting I across all sample sizes. Under setting II, our method produces much less biased estimates, with improvements becoming more evident as the sample size increases. In Table S1 (Appendix 4), we further compare the model performance around the peak of these two risk curves. Although the MAPE becomes larger around the peak in both methods, our method still provides less biased predictions. Figure S1 (Appendix 4) shows curve‐fitting results for the exposure effects averaged over replicates in Settings I and II, with shaded areas representing intervals of 95% predictions. Our method tends to generate less biased estimates, especially around the peaks. Across both settings, however, the proposed method tends to exhibit a larger standard deviation than the conventional spline‐based SCCS method for all sample sizes, reflecting the trade‐off between flexibility and stability in sparse‐event settings. These additional simulations (*n* = 200 and 500) highlight the finite‐sample behavior of flexible curve estimation. When the number of cases is small (*n* = 200), both spline‐based approaches exhibit increased variability and reduced accuracy, consistent with limited information to support stable estimation of the risk curve. As the sample size increases, estimation stabilizes and the advantage of the proposed approach becomes clearer for the more complex PK/PD shaped trajectory in Setting II.

**TABLE 1:** Simulation results for exposure‐related relative incidence in single exposure settings I and II.

					Mean estimates (SD)			CP		
Setting	*N*	Method	MISE (SD)	MAPE (SD)	$\hat{\theta }$_1_	$\hat{\theta }$_2_	$\hat{\theta }$_3_	$\hat{\theta }$_1_	$\hat{\theta }$_2_	$\hat{\theta }$_3_
I	200	PLSI‐SCCS	5.320 (1.560)	0.582 (0.152)	0.398 (0.265)	0.798 (0.242)	1.206 (0.230)	0.965	0.965	0.950
		Spline‐based SCCS	5.217 (0.454)	0.563 (0.152)	0.419 (0.261)	0.807 (0.234)	1.212 (0.228)	0.965	0.970	0.965
	500	PLSI‐SCCS	3.445 (0.950)	0.366 (0.097)	0.398 (0.154)	0.794 (0.157)	1.183 (0.158)	0.965	0.950	0.935
		Spline‐based SCCS	3.327 (1.009)	0.366 (0.093)	0.401 (0.141)	0.786 (0.152)	1.190 (0.154)	0.975	0.945	0.940
	1000	PLSI‐SCCS	2.538 (0.748)	0.273 (0.080)	0.398 (0.115)	0.796 (0.108)	1.191 (0.116)	0.970	0.960	0.925
		Spline‐based SCCS	2.669 (0.676)	0.281 (0.068)	0.398 (0.112)	0.788 (0.110)	1.192 (0.107)	0.955	0.925	0.940
II	200	PLSI‐SCCS	5.389 (1.855)	0.560 (0.172)	0.388 (0.268)	0.805 (0.235)	1.212 (0.241)	0.955	0.970	0.955
		Spline‐based SCCS	5.665 (1.101)	0.546 (0.137)	0.371 (0.285)	0.795 (0.237)	1.205 (0.245)	0.945	0.965	0.945
	500	PLSI‐SCCS	3.359 (1.298)	0.359 (0.128)	0.403 (0.175)	0.801 (0.163)	1.201 (0.162)	0.930	0.945	0.940
		Spline‐based SCCS	4.945 (0.615)	0.432 (0.073)	0.387 (0.181)	0.791 (0.163)	1.193 (0.157)	0.930	0.935	0.935
	1000	PLSI‐SCCS	2.481 (0.802)	0.259 (0.084)	0.400 (0.128)	0.804 (0.115)	1.204 (0.107)	0.930	0.940	0.945
		Spline‐based SCCS	4.553 (0.846)	0.351 (0.058)	0.389 (0.128)	0.801 (0.109)	1.201 (0.106)	0.910	0.970	0.945

*Note*: ‘MISE’: median integrated squared error. ‘MAPE’: mean absolute predicted error. ‘CP’: coverage probability. $\theta_{1},\theta_{2}$ and $\theta_{3}$ denote the log–relative incidence parameters associated with the time‐varying confounder (age groups), with the first age group serving as the reference category. For each simulation setting, 200 independent replicate datasets were generated. Reported mean estimates and standard deviations are the empirical mean and standard deviation across the 200 replicates. Setting I: Bell shaped risk curve $R_{1}\left(t\right)$. Setting II: PK/PD shaped risk curve $R_{2}\left(t\right)$.

We further evaluated the performance of our method under multiple exposure settings III to VI. As demonstrated in Table 2, our model consistently performs well under all these settings in estimating age effects (θs). MISE_1_ and MISE_2_ in Table 2 denote the MISE values of model predictions for individual exposure effects, assuming no overlap between exposures. As noted in Section 3.1, the first risk curves used in settings III‐V are the bell shaped curve used in setting I. Similarly, the risk curves in setting VI and the second curves in settings III‐V are the PK/PD shaped curve used in setting II. As shown in Table 2, the MISE values of these single curves are close to MISE values obtained under single exposure settings in Table 1, indicating that the single exposure effects are not biasedly estimated in multiple‐exposure settings, even with overlapping risk periods (settings IV‐VI). Figure S2 (Appendix 4) presents model predictions for exposure effects when risk periods do not overlap (Setting III), showing that in this simple setting both our method and the standard SCCS method are capable of capturing the time‐varying nature of exposure effects.

**TABLE 2:** Simulation results for exposure‐related relative incidence in multiple exposure settings III to VI.

				Mean estimates (SD)			CP		
Setting	MISE_1_ (SD)	MISE_2_ (SD)	MAPE (SD)	$\hat{\theta }$_1_	$\hat{\theta }$_2_	$\hat{\theta }$_3_	$\hat{\theta }$_1_	$\hat{\theta }$_2_	$\hat{\theta }$_3_
III	2.598 (0.763)	2.847 (0.890)	0.280 (0.062)	0.401 (0.110)	0.801 (0.109)	1.200 (0.110)	0.960	0.955	0.960
IV	2.792 (0.833)	2.679 (0.879)	0.287 (0.085)	0.394 (0.114)	0.803 (0.115)	1.205 (0.102)	0.975	0.945	0.955
V	2.816 (0.848)	2.819 (0.839)	0.317 (0.067)	0.400 (0.113)	0.803 (0.098)	1.200 (0.097)	0.950	0.975	0.970
VI	2.457 (0.685)	—	0.274 (0.075)	0.415 (0.115)	0.815 (0.104)	1.222 (0.101)	0.960	0.970	0.975

*Note*: ‘MISE’: median integrated squared error. MISE_1_ and MISE_2_ indicate MISE for the first and second risk period respectively, assuming there is no overlapping. ‘MAPE’: mean absolute predicted error. ‘CP’: coverage probability. $\theta_{1},\theta_{2}$ and $\theta_{3}$ denote the log–relative incidence parameters associated with the time‐varying confounder (age groups), with the first age group serving as the reference category. For each simulation setting, 200 independent replicate datasets were generated. Reported mean estimates and standard deviations are the empirical mean and standard deviation across the 200 replicates. Setting III: Two non‐overlapping risk periods; bell shaped $R_{1}\left(t\right)$ and PK/PD shaped $R_{2}\left(t\right)$. Setting IV: Overlapping risk periods; bell shaped $R_{1}\left(t\right)$ and constant $R_{3}\left(t\right)$. Setting V: Overlapping risk periods; bell shaped $R_{1}\left(t\right)$ and PK/PD shaped $R_{2}\left(t\right)$. Setting VI: Repeated and overlapping risk periods; two PK/PD shaped $R_{2}\left(t\right)$. For setting IV and V, model prediction based on overall exposure effects by assuming the second risk period starts 28 days after the first risk period. For setting VI, model predictions based on assuming the second risk period starts 14 days after the first risk period. The sample size is *n* = 1000 for all settings.

**FIGURE 2:**
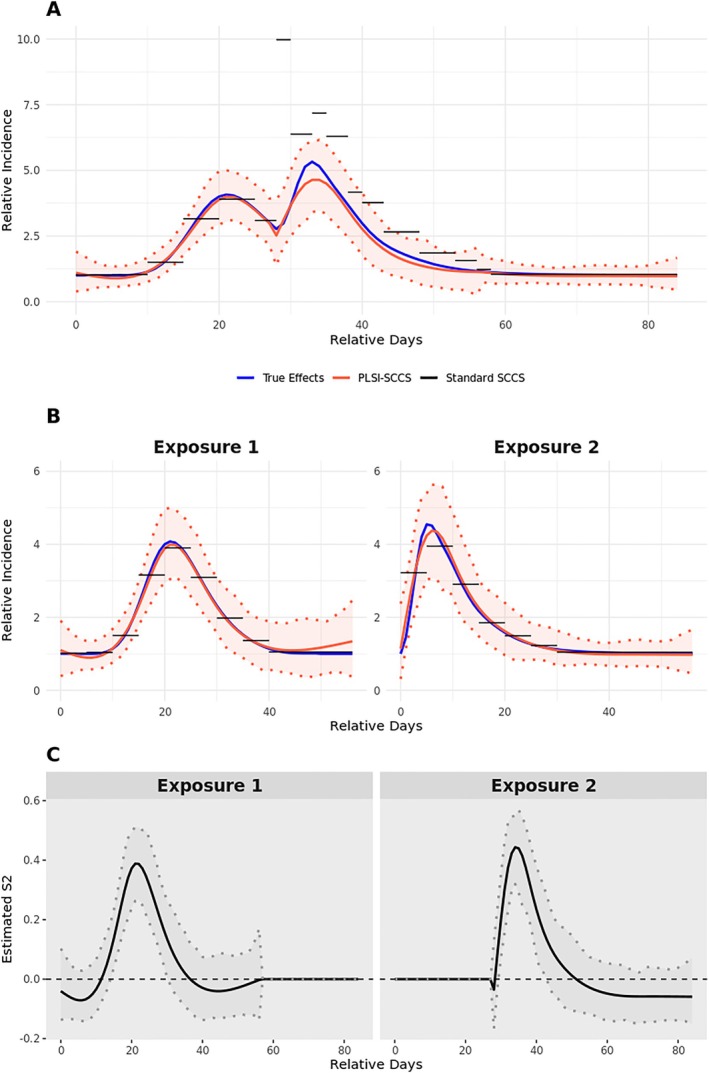
Predicted relative incidence curves and exposure contributions in simulation setting V. Multiple exposure setting with overlapping risk periods; bell shaped $R_{1}\left(t\right)$ followed by PK/PD shaped $R_{2}\left(t\right)$ separated by uniform (21, 56) days. Panel (A) displays the overall prediction for exposure‐related relative incidence (RI), assuming that the second risk period begins 28 days after the start of the first risk period. Panel (B) predicts exposure‐related RI for each risk period. Panel (C) shows the estimated ${\hat{S}}_{2}$ curves for the relative importance of each exposure when the overlapping risk period begins 28 days after the first. For panel (C), ${\hat{S}}_{2}$ of each replicate is multiplied by the sign of its maximum absolute value to ensure that the first exposure is visualized as positive. The shaded area covers the 95% prediction interval across all replicates. Details on how the step‐function interval lengths were chosen for the standard SCCS are provided in Appendix 3.

**FIGURE 3:**
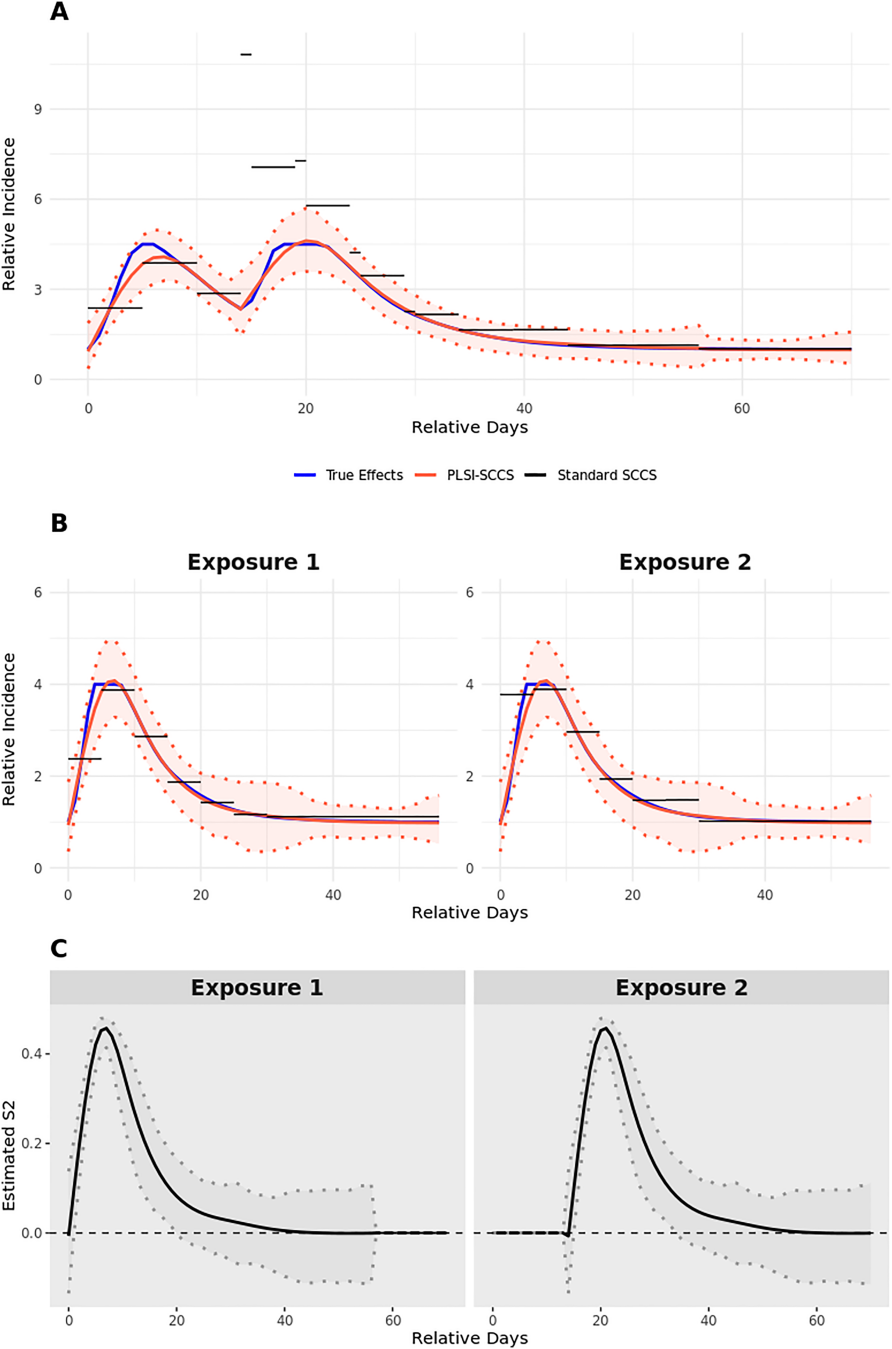
Predicted relative incidence curves and exposure contributions in simulation setting VI. Multiple exposure setting with overlapping risk periods; The risk is two PK/PD shaped curves $R_{2}\left(t\right)$ separated by uniform (14, 28) days. Panel (A) displays the overall prediction for exposure‐related relative incidence (RI), assuming that the second risk period begins 14 days after the start of the first risk period. Panel (B) predicts exposure‐related RI for each risk period. Panel (C) shows the estimated ${\hat{S}}_{2}$ curves for the relative importance of each exposure when the overlapping risk period begins 14 days after the first. For panel (C), we multiply ${\hat{S}}_{2}$ of each replicate by the sign of its maximum absolute value to ensure that the first exposure is visualized as positive. The shaded area covers the 95% prediction interval across all replicates. Details on how the step‐function interval lengths were chosen for the standard SCCS are provided in Appendix 2.

For settings with overlapping risks (i.e., Settings IV to VI), the true overall exposure effects during the observation period vary across cases, depending on when the second risk period begins. Therefore, we presented overall predictions at a fixed start time for the second risk period to evaluate the predictions for the overlapping risk interval. Specifically, for Settings IV and V, we examined predictions when the second risk period starts 28 days after the first. For Setting VI, we focused on predictions when the second risk period starts 21 days after the first. Figure S3 (Appendix 4) and Figures 2 and 3 present the model predictions for Settings IV and VI, respectively. As illustrated in Panel A of all these figures, our method effectively captures changes in overall exposure effects during the overlapping period for all three scenarios. For setting IV, where there is a multiplicative relationship between exposures, the proposed method slightly underestimate the overall effect around the peak while the standard SCCS method slightly overestimates it (Panel A in Figure S3). However, when there is an additive relationship, estimates from the standard SCCS method can be severely biased for overlapping periods (Panel A in Figures 2 and 3). Panel B presents model predictions on individual exposure effects. Our method continues to accurately capture time‐varying effects of individual exposures. Meanwhile, the standard SCCS method may produce biased estimates of the effects of individual exposures when there is an additive relationship (see the first 5 days of the second risk period of Panel B in Figure 3). Estimates of the ${\hat{S}}_{2}$ curves in panel C provide a visual interpretation of each exposure's relative effects within the overlapping risk period. In these figures, the ${\hat{S}}_{2}$ curves have a similar shape as true exposure effects, indicating a monotonic relationship between single exposure effects and overall effects during the overlapping period. That is, when one exposure exhibits a larger effect, the overall effect increases correspondingly, and the ${\hat{S}}_{2}$ curves will present a larger relative importance of this exposure. Additional runtime comparisons are provided in Appendix 4, Table S2. Under the single‐exposure setting, PLSI‐SCCS required substantially longer computation time than the conventional spline‐based SCCS method. Computation time increased with sample size and with the numbers of basis functions $M_{1}$ and $M_{2}$. In the multiple‐exposure setting, a single model fit typically required several minutes.

## 4 Application

In this section, we illustrate our method by applying it to two real datasets. They are chosen for illustration of two scenarios: exposures to two treatments, each with different RI functions, and repeated doses of the same treatment with the same RI function each dose. 95% CIs are obtained from 400 bootstrap samples.

### 4.1 Hib, MMR and Febrile Convulsions

**FIGURE 4:**
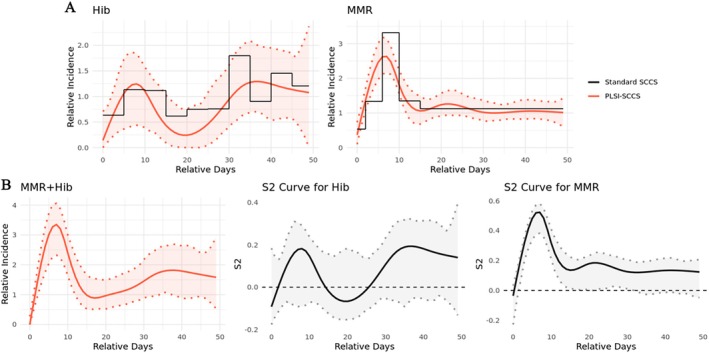
Predicted relative incidence curves and exposure contributions for Hib, MMR vaccinations, and febrile convulsions. Panel (A) presents exposure‐related RI for Hib and MMR vaccinations, respectively. Details on how the step‐function interval lengths were chosen for the standard SCCS are provided in Appendix 3. Panel (B) presents the predicted risk curve for the overlapping period when MMR and Hib vaccinations were administered on the same day, as well as ${\hat{S}}_{2}$ curves for each vaccination separately.

We analyzed a dataset on Hib vaccination, MMR vaccination, and febrile convulsions among children aged from 366 to 730 days, which is included in the ‘SCCS’ R package [24]. The Hib vaccine is typically administered during the first year after birth, with a booster dose given during the second year of life. However, the MMR vaccine, which is suspected to increase the risk of convulsions, is also administered in the second year. Therefore, to assess the possible effect of the Hib booster vaccine on febrile convulsions, it is essential to account for the MMR vaccine, as both may be given simultaneously or in close succession. In the dataset, 2435 convulsions were recorded among 2201 children. Of these, 542 (24.63%) children received the Hib booster dose, and 2031 (92.28%) received the MMR vaccine. A total of 212 (9.63%) and 314 (14.27%) children received the Hib booster and MMR vaccines on the same day or different days, respectively. The mean (SD) values of age at receiving the Hib booster dose and MMR vaccine were 518 (116) days and 433 (75) days, respectively. Previous studies indicate that MMR vaccination had a risk period spanning 2 to 5 weeks for febrile convulsion [35, 36]. Accordingly, we chose a 50‐day risk window for both Hib and MMR vaccines, which should adequately capture the true risk window. In fitting the PLSI‐SCCS model, we followed the estimation procedure described in Section 2.4, using $M_{1}$ = 10 knots to approximate the single‐index link function and selecting the best smoothing parameter λ and $M_{2}$ knots using the cross‐validation method. The best $M_{2}$ selected is seven with λ = 0.64. The standard SCCS model was fitted with risk periods for Hib vaccination were represented by step functions at 5‐day interval and that for MMR were defined by breaks at 2, 6, 10, and 15 days. We defined 12 age groups with an interval of 4 weeks to represent age‐related RI function in the analysis. Figure 4 illustrates the model predictions for the RI functions for Hib and MMR vaccinations. Panel A presents model predictions and 95% CIs for the RI curve of Hib and MMR vaccinations for cases that did not have overlapping risk periods (the two vaccination dates differ by at least 50 days). We further compare these predictions to results from standard SCCS models with the same age group definitions. Black lines in the figure represent estimates from the standard SCCS method. Detailed results from the standard SCCS method are provided in Table S3 (Appendix 4). Panel B illustrates the predicted RI curve for the overlapping period when MMR and Hib were administered on the same day, along with ${\hat{S}}_{2}$ curves for each vaccine.

Panel A in Figure 4 shows a significant increase in the risk of febrile convulsions from 2 to 12 days following MMR vaccination, peaking at day 8. The incidence at this peak was ˜2.5 times higher than the incidence during the baseline period before and after the risk period, with the age effect held constant. The risk period after Hib vaccination does not show a significantly increased risk of febrile convulsions. These findings are consistent with analyses in previous studies [2]. As shown in Panel B, during the overlapping risk period, the co‐administration of Hib and MMR vaccines appears to result in a higher peak at day 8, indicating an incidence of febrile convulsions about three times higher than normal. Additionally, we observed a significantly lower risk of febrile convulsions at day 0 for overlapping periods, which may be due to a common practice that children with fever are told to delay vaccination, as indicated by no convulsion reported on the day of co‐administration for both vaccines in this dataset (See Figure S4, Appendix 4). As shown by the ${\hat{S}}_{2}$ curves in Panel B, the increased risk in the overlapping period is mainly contributed by the MMR vaccination, while the Hib vaccination plays a minor role throughout this period, with an insignificant contribution to the overall effect. According to Figure S5 (Appendix 4), the increase of ${\hat{S}}_{2}$ values for each exposure is always associated with the increase of the risk of febrile convulsions during the overlapping period.

### 4.2 Malaria Prevention Trial

While SCCS is often used to study drug and vaccine safety, it can also be used to study their efficacy [2, 3, 4, 5, 6, 7, 8]. We employed data from a randomized placebo‐controlled trial on malaria chemoprevention in Ghana [37] as an illustrative example of a repeated‐dose study. Infants were recruited from immunization clinics at ˜2 months of age and followed for up to 24 months to monitor episodes of clinical malaria. Participants were randomized to receive four doses of either placebo or SP at around 1, 2, 7, and 10 months post‐enrollment. The primary endpoint was defined as a healthcare visit with microscopy‐confirmed malaria parasites, accompanied by a measured temperature of at least 37.5°C or a parental report of fever. To avoid double‐counting, malaria episodes within 7 days of a previous episode in the same infant were excluded [38].

Although unexposed subjects do not directly provide information about the exposure under the SCCS framework, including them can improve the precision of estimating the exposure effect [39]. Therefore, our SCCS analysis included individuals who had experienced the adverse event (i.e., clinical malaria) from both the SP arm and the placebo‐controlled arm, which included 1271 infants and 2019 recorded malaria episodes. Of these, 603 infants and 961 malaria episodes were in the SP arm. The four doses were administered at a mean (SD) of 32 (13), 70 (38), 236 (37), and 324 (28) days, respectively. Previous studies suggest that the terminal elimination half‐life of SP is 1–3 weeks but clinical efficacy persisting for up to about 6–8 weeks [38, 40, 41, 42]. Therefore, overlapping exposures primarily exist in the first two doses. In this analysis, we used a long window of 140 days to fully capture the effective period of SP doses. Following the estimation procedure in Section 2.4, we fit the PLSI‐SCCS model under the assumption of the same RI function for each dose. With $M_{1}$ = 10, we selected $M_{2}$ = 4 and λ = 0.14 through cross‐validation. We also included 13 age groups (12 two‐month intervals for children under 2 years of age, plus one interval for 2 years or above) and an indicator for the rainy season (July–November; Dry season otherwise) to account for time‐varying covariates.

**FIGURE 5:**
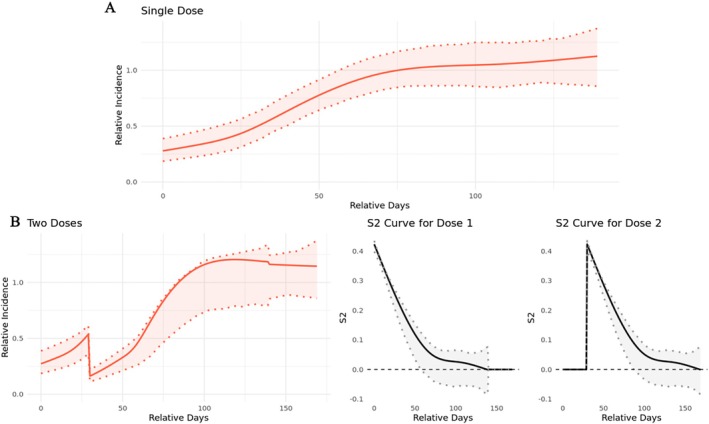
Predicted relative incidence curves and exposure contributions for the malaria chemoprevention study. Panel (A) presents exposure‐related RI for a single SP dose. Panel (B) presents the predicted risk curve and ${\hat{S}}_{2}$ curves for the overlapping period when the second dose was administered at day 30.

Figure 5 presents the estimated effects of the single SP dose and repeated doses. Panel A shows the trajectory of the protective efficacy of a single dose. Panel B presents model predictions when the second dose is administered 30 days after the first, along with the S_2_ curves for both doses. When initially taken, a single SP dose can lead to an RI of clinical malaria of 0.27, when age and season effects are fixed. The protective effect (one minus RI) then slowly decreases and becomes insignificant around day 50. Repeated doses can extend the effective period. Specifically, the second dose at day 30 after dose 1 resulted in a RI of 0.16 and became insignificant around 50 days after administration. In this analysis, we assumed the same RI curve across all doses. Thus, the S_2_ curves for doses 1 and 2 are identical. However, we can still compare the relative importance of these two doses at a specific time by comparing the magnitudes of the $\hat{S}$_2_ curves. When two repeated doses are administered, the latter dose, which has a larger $\hat{S}$_2_ value, always plays a major role during the overlapping period. As presented in Figure S5 (Appendix 4), when $\hat{S}$_2_ increases, the RI of clinical malaria always decreases, indicating a larger protective effect.

## 5 Conclusion and Discussion

Multiple exposures that may involve multiple drugs or vaccines or repeated dosing regimens are common in medicine. As exposure effects can vary over time, standard SCCS analysis using a step function usually requires predefined exposed periods and interaction terms based on hypotheses or prior biological knowledge. However, previous studies have shown that different specifications of exposed periods can yield markedly different results [12], and in the absence of prior knowledge, more flexible methods for investigation of the trajectory of treatment effect are generally recommended [43, 44]. Although the SCCS design is a popular alternative to cohort and case–control methods, it currently lacks a flexible approach for multiple‐exposure scenarios. To address this gap, we propose the PLSI‐SCCS method, which is semiparametric and partially linear. In its framework, a nonlinear single‐index function captures exposure effects as well as time‐varying interactions, while the effects of other time‐varying confounders are captured by step functions.

Our method offers several advantages over existing spline‐based SCCS methods. First and foremost, it relaxes the non‐overlapping risk period assumption [13], thereby enabling applications in more general multiple‐exposure settings. Simulations in our study have shown that the PLSI‐SCCS method performs comparably to conventional spline‐based SCCS methods when applied to single‐exposure scenarios, yet accommodates complex overlapping exposures. Second, by consolidating spline functions into a single index, the PLSI‐SCCS estimates interactive effects in a parsimonious manner. This consolidation also yields a graphically interpretable representation of each exposure's relative importance in overlapping risk windows. Applications on the Hib and MMR vaccines study and malaria chemoprevention study further illustrated these benefits. Compared with the three‐part SCCS model for overlapping risk periods [19], which emphasizes interpretability via prespecified intervals and piecewise constant risk terms, our approach provides a complementary alternative that accommodates smooth time‐varying exposure and overlap effects in multiple‐exposure settings.

A limitation of the proposed method is its higher computational cost relative to existing SCCS approaches. This reflects the added flexibility of the model, particularly for handling multiple exposures with overlapping risk periods and time‐varying interaction patterns. In applications with only a single exposure, or when such flexibility is not required for multiple exposure estimation, conventional spline‐based SCCS models may be preferable. As repeated model fits such as bootstrap resamples can be run independently, the running time may be reduced through parallel implementation.

Several key considerations are warranted to appropriately employ the PLSI‐SCCS model. Firstly, as with other spline‐based SCCS methods, the specification of the risk periods $\left(c_{k},d_{k}\right]$ is flexible. When the true risk period is indefinite or long‐lasting, $d_{k}$ can be set to the time at the end of the observation period to fully capture the exposure effects. However, including long risk periods may increase confounding with time‐varying covariates such as age and season. To address this issue, we suggest including unexposed cases in the data analysis, which does not involve confounding between exposure and time‐varying covariate and improves the estimation of exposure effects [39]. Secondly, it is important to note that using very long nominal risk periods is not the optimal choice for all data analyses. Since spline knots are determined within the risk period, an appropriate specification of $d_{k}$ ensures that most knots are efficiently used to approximate the true exposure period. If the risk period is overly extended, some knots may become redundant, increasing the complexity of our model and potentially leading to overfitting of the unexposed periods while inadequately fitting the true risk periods. Thirdly, the use of PLSI‐SCCS method requires careful specification of constraints to ensure the identifiability, failure to enforce the unit‐norm and sign constraints on can lead to non‐unique parameterizations (scale and sign switching) in which substantially different‐looking S_2_ curves correspond to essentially the same fitted relative incidence curves. This typically manifests as failed convergence of algorithm, numerical instability and inconsistent directional interpretation across model refits. Lastly, PLSI‐SCCS estimates flexible smooth functions including overall and exposure‐specific components. As a result, stable estimation of the RI and S_2_ curves may require a sufficient number of events distributed across relative time within the risk window. In vaccine safety studies targeting rare and severe adverse events, where case counts are often limited or the available information within the risk periods is sparse, users may consider employing stronger smoothness penalization, using fewer spline basis functions/knots and conducting sensitivity analyses against more parsimonious SCCS specifications to assess robustness of model estimation. No universal threshold is recommended, as the appropriate choice depends on the complexity of the exposure‐risk relationship and the information available in the data.

Although our method is motivated by and developed based on the SCCS design, it is a specific application of the PLSI method. PLSI models are commonly used in environmental epidemiology studies to approximate the cumulative effects of time‐dependent exposure mixtures. In our approach, overlapping risk periods are treated as exposure “mixtures” with time‐varying effects, while non‐overlapping periods are simply times when other “mixture components” are absent. As with other PLSI methods, caution is required when interpreting results. Specifically, due to the identifiability constraint, single‐index coefficients (i.e., S_2_ curves) only indicate the relative direction and importance of each exposure [31]. The interpretation of exposure effects within the overlapping period should consider both the single‐index coefficients and the form of the single‐index link function. Only when the single index link function is monotone, S_2_ curves can have a general interpretation as “effect”. Otherwise, the direction of these effects can only be interpreted when all S_2_ values at the same time point are given [25, 32].

In real world safety studies with multiple exposures or repeated doses, it is of interest to determine a dosing interval that achieves the desired effect while keeping any overlap‐related elevation in risk at an acceptable level. While we do not introduce a dedicated co‐administration indicator, co‐administration (including repeat doses of the same vaccine) is accommodated through the joint single index, so that overlapping risk periods are reflected in the predicted RI curve and the corresponding S_2_ curves. Practically, after fitting the model, one can generate model‐based predictions of the overall RI curve under prespecified dosing intervals by shifting the dose 2 exposure window relative to dose 1 and recomputing the predicted RI trajectory. When the predicted RI attributable to dose 1 has returned close to baseline by the time dose 2 is administered and the S_2_ curve of dose 1 remains near its unexposed level over the overlap period, additional excess risk attributable to dose 1 is less likely. When overlap cannot be avoided, the model can still be used to compare candidate dose intervals by examining predicted RI over the overlap window, together with S_2_ curves that indicate which dose dominates the joint effect during overlap. For instance, the S_2_ curve for Hib in Figure 4 remains close to its unexposed level over the relevant overlap period, and the S_2_ contribution of dose 1 in Figure 5 becomes small at later times (e.g., after ˜50 days), suggesting limited contribution of the earlier exposure during overlap in those periods. Nevertheless, we emphasize that the primary objective of the proposed method is to provide stable estimation and graphical interpretation of exposure effects in the presence of overlapping risk periods, rather than to identify an optimal vaccination interval, which would also depend on immunogenicity and implementation considerations [45].

Future research in this direction can focus on the following points. First, the PLSI‐SCCS method consolidates exposure effects into a single index, which may result in a loss of information when many risk periods overlap. Future research can explore alternative methods to extend the single‐index link function to multiple‐index link functions, in order to find a balance between model parsimony and prediction performance. Second, while we currently represent age effects and other time‐varying confounders with step functions, the approach can readily be extended to accommodate spline‐based terms for these time‐varying effects. Finally, investigating interactions between age and exposures in a spline‐based framework may further illuminate the complexity of multiple‐exposure patterns.

## Funding

The authors have nothing to report.

## Conflicts of Interest

The authors declare no conflicts of interest.

## Supporting information


**Appendix S1:** Supporting Information.

## Data Availability

Sharing of the data is not applicable to this article as no new data were created or analyzed in this study. Professor D. Chandramohan, the corresponding author of the cluster‐randomized trial, granted us permission to use—but not distribute—the malaria prevention trial data. Access to the data may be available from Professor D. Chandramohan upon reasonable request and with permission. R codes for model, estimation and simulation are available at https://github.com/Xuezhixing‐Zhang/PLSI‐SCCS.
