# Supplementary material for: Flexible and Interpretable Modeling of Overlapping Exposure Risks in Self‐Controlled Case Series Analysis
Source: Stat Med. 2026 Apr 14;45:e70552. doi: 10.1002/sim.70552 (PMC13078252; doi:10.1002/sim.70552)

Appendix 1: Details of Constraints for the PLSI-SCCS model.

| Constraint | Form | Objective | Consequences of violation |
| --- | --- | --- | --- |
| Nonnegativity of $\text{S}_{\text{1}}\text{(}\text{u}\text{)}$ | $S_{1}(u)\approx\boldsymbol{B}_{1}^{T}(u)\beta^{2}=\sum_{l=1}^{M_{1}} \beta_{l}^{2}B_{1l}^{T}(u)$ | Ensures $\text{S}_{\text{1}}\text{(}\text{u}\text{)}\text{ }\text{≥}\text{ 0 }$so that $\text{S}_{\text{1}}\text{(}\text{u}\text{)}$ behaves like an RI function. | If nonnegativity not enforced, $\text{S}_{\text{1}}$ can oscillate and become negative, making it unsuitable for use as an RI function. |
| Unit norm constraint on ***γ*** | $\vert\vert\gamma\vert\vert^{2}=\sqrt{\gamma_{11}^{2}+...+\gamma_{pM_{2p}}^{2}}=1$ | Fixes scale non-identifiability of the single-index. Without it, any rescaling of***γ*** can be absorbed by the nonparametric link function and thus cannot be identified. This constraint also makes***γ***’s magnitude interpretable only relatively. | Without this constraint,***γ*** and the nonparametric link function can trade-off the scale, leading to multiple solutions of***γ***. |
| Direction constraint on ***γ*** | $\gamma_{11}>0$ | Fixes the sign-flip ambiguity that can be caused by redefining the link function. | Without this constraint,***γ*** can have multiple solutions as the sign can be absorbed by the link function. |
| Anchor-point constraint on $\text{S}_{\text{1}}\text{(}\text{u}\text{)}$ | $\text{S}_{\text{1}}\text{(}\text{0}\text{)}$ = 1 for unexposed periods | The typical constraint for SCCS design. It sets the reference level for unexposed periods as 1. | Without anchoring, you can multiply $\text{S}_{\text{1}}\text{(}\text{u}\text{)}$ by a constant and divide another component correspondingly with no change in fit. Baseline and exposure functions become non-unique; interpretation of absolute levels becomes arbitrary. |
| Anchor-point constraint on $\text{S}_{\text{2}\text{k}}\text{(}\text{t}\text{−}\text{c}\text{)}$ | $\text{S}_{\text{2}\text{k}}\text{(}\text{t}\text{−}\text{c}\text{)}$ = 0 for the unexposed period of exposure $k$ | Ensures S_2_ curves reflect relative changes only. It defines what “zero effect” means for each exposure k: the curve is interpreted as deviation from the unexposed period. | Without anchoring, all  $\text{S}_{\text{2}}$ curves can shift together by the same function (or constant) while another term compensates. |

#### Appendix 2: Inference on θ with PLSI-SCCS model.

The log-likelihood function given in Section 2.4 is

$$\begin{matrix} l^{c}(\boldsymbol{\lambda},\boldsymbol{\beta},\boldsymbol{\theta}) & =\sum_{i=1}^{N} \sum_{j=1}^{n_{i}} \left\{ \boldsymbol{z}_{i}\left( t_{ij} \right)^{T}\boldsymbol{\theta}+log\left\{ \boldsymbol{B}_{1}^{T}\left[ \left( \boldsymbol{B}_{2}\boldsymbol{R}_{i}\left( t_{ij} \right) \right)^{T}\boldsymbol{\gamma} \right]\boldsymbol{\beta}^{2} \right\} \right. \\ & -\sum_{i=1}^{N} n_{i}log\left\{ \int_{a_{i}}^{b_{i}} exp\left( \boldsymbol{z}_{i}(t)^{T}\boldsymbol{\theta} \right)\left\{ \boldsymbol{B}_{1}^{T}\left[ \left( \boldsymbol{B}_{2}\boldsymbol{R}_{i}(t) \right)^{T}\boldsymbol{\gamma} \right]\boldsymbol{\beta}^{2} \right\}dt \right\} \end{matrix}$$

With the penalty term, we have the objective function:

$$\begin{matrix} Q(\boldsymbol{\gamma},\boldsymbol{\beta},\boldsymbol{\theta}) & =l^{c}(\boldsymbol{\beta},\boldsymbol{\theta})-\gamma\int\left( \sum_{l=1}^{m_{1}} \beta_{l}^{2}B_{1l}^{''}(u) \right)^{2}du \\ & =l^{c}(\boldsymbol{\beta},\boldsymbol{\theta})-\lambda\left( \left( \boldsymbol{\beta}^{2} \right)^{T}\boldsymbol{D}\boldsymbol{\beta}^{2} \right) \end{matrix}$$

The first order derivative for $\boldsymbol{\theta}$ is:

$$\frac{\partial Q(\boldsymbol{\gamma},\boldsymbol{\beta},\boldsymbol{\theta})}{\partial\boldsymbol{\theta}}=\sum_{i=1}^{N} \left( \sum_{j=1}^{n_{i}} \boldsymbol{z}_{i}\left( t_{ij} \right)-n_{i}\frac{\int_{a_{i}}^{b_{i}} \boldsymbol{z}_{i}(t)exp\left( \boldsymbol{z}_{i}(t)^{T}\boldsymbol{\theta} \right)k_{i}(t)dt}{\int_{a_{i}}^{b_{i}} exp\left( \boldsymbol{z}_{i}(t)^{T}\boldsymbol{\theta} \right)k_{i}(t)dt} \right)$$

where $k_{i}(t)=\boldsymbol{B}_{1}^{T}\left[ \left( \boldsymbol{B}_{2}\boldsymbol{R}_{i}(t) \right)^{T}\boldsymbol{\gamma} \right]\boldsymbol{\beta}^{2}$, which is a numeric value dependent on time $t$.
Let $\boldsymbol{S}_{i}=\sum_{j=1}^{n_{i}} \boldsymbol{z}_{i}\left( t_{ij} \right)$ be the sum of all $\boldsymbol{Z}_{i}(t)$ for all $n_{i}$ events, $C_{i}=\int_{a_{i}}^{b_{i}} exp\left( \boldsymbol{z}_{i}(t)^{T}\boldsymbol{\theta} \right)k_{i}(t)dt$ be the denominator and $\boldsymbol{E}_{i}=\frac{\int_{a_{i}}^{b_{i}} \boldsymbol{z}_{i}(t)exp\left( \boldsymbol{z}_{i}(t)^{T}\boldsymbol{\theta} \right)k_{i}(t)dt}{\int_{a_{i}}^{b_{i}} exp\left( \boldsymbol{z}_{i}(t)^{T}\boldsymbol{\theta} \right)k_{i}(t)dt}$. Then the score function can be rewritten as:

$$\frac{\partial l^{c}(\boldsymbol{\gamma},\boldsymbol{\beta},\boldsymbol{\theta})}{\partial\boldsymbol{\theta}}=\sum_{i=1}^{N} \left( \boldsymbol{S}_{i}-n_{i}\boldsymbol{E}_{i} \right)$$

The second order derivative is

$$\begin{matrix} \frac{\partial l^{c}(\boldsymbol{\gamma},\boldsymbol{\beta},\boldsymbol{\theta})}{\partial\boldsymbol{\theta}\partial\boldsymbol{\theta}^{T}} & =-\sum_{i=1}^{N} n_{i}\frac{\partial\boldsymbol{E}_{i}}{\partial\boldsymbol{\theta}^{T}} \\ & =-\sum_{i=1}^{N} n_{i}\left( \frac{\int_{a_{i}}^{b_{i}} \boldsymbol{Z}_{i}(t)\boldsymbol{Z}_{i}(t)^{T}exp\left( \boldsymbol{Z}_{i}(t)^{T}\boldsymbol{\theta} \right)k_{i}(t)dt}{C_{i}}-\boldsymbol{E}_{i}\boldsymbol{E}_{i}^{T} \right) \end{matrix}$$

Let $\boldsymbol{V}_{i}=\frac{\partial\boldsymbol{E}_{i}}{\partial\boldsymbol{\theta}^{T}}$. Then

$$\begin{matrix} \frac{\partial l^{c}(\boldsymbol{\gamma},\boldsymbol{\beta},\boldsymbol{\theta})}{\partial\boldsymbol{\theta}\partial\boldsymbol{\theta}^{T}} & =-\sum_{i=1}^{N} n_{i}\boldsymbol{V}_{i} \\ I(\boldsymbol{\theta}) & =\sum_{i=1}^{N} n_{i}\boldsymbol{V}_{i} \\ Var(\boldsymbol{\theta}) & =I(\boldsymbol{\theta})^{-1} \end{matrix}$$

The standard error is

$$SE(\boldsymbol{\theta})=\sqrt{diag(Var(\boldsymbol{\theta}))}$$

The $95\%$ confidence interval for $\theta_{k}$ is

$$\theta_{k}\pm z_{0.975}\times SE\left( \theta_{k} \right)$$

**Appendix 3: Simulation Settings and Evaluation Methods.** R_1_(t): bell shape risk curve of 50 days. R_2_(t): concentration-time shaped PK/PD curve of 50 days. R_3_(t): constant risk of 29 days. c_1_, c_2_ and c_3_ refer to the start time for risk periods R_1_(t), R_2_(t) and R_3_(t), respectively. When fitting the model, the nominal risk periods for R_1_(t) and R_2_(t) were extended by seven days to better reflect real-world scenarios, where using a longer risk period may more accurately capture the true risk period.

| Setting | RI | Risk Periods | Evaluation Method |
| --- | --- | --- | --- |
| I | RI(t) = R_1_(t - c_1_) | The start time of R_1_(t)/R_2_(t) was uniformly randomly drawn from the observation period. | Results from the PLSI-SCCS method are compared to the spline-based SCCS method in R 'SCCS' package, using the function 'smoothexposccs'. |
| II | RI(t) = R_2_(t - c_2_) |  |  |
| III | RI(t) = R_1_(t - c_1_) or R_2_(t - c_2_) | The start time of R_1_(t) was randomly drawn from the observation period. The start time of R_2_(t) was set to 56 days after the start time of R_1_(t). | As current spline-based SCCS functions in R are not designed for multiple exposures. We compared results from the PLSI-SCCS method to those from the standard SCCS method using the R 'SCCS' package with the function 'standardsccs'. In fitting the standard model, we divided the risk period R_1_(t) into nine intervals with breaks at days 0, 5, 10, 15, 20, 25, 30, 35, and 40. The risk period R_2_(t) was segmented into seven intervals with breaks at days 0, 5, 10, 15, 20, 25, and 30. For settings IV to VI, where risk periods overlap, we included interaction terms in the model. Specifically, for Setting VI, we fit the standard SCCS model with varying RI functions, as the current 'SCCS' package does not support interactions between exposures that share the same RI function. |
| IV | RI(t) = R_1_(t - c_1_)R_3_(t - c_3_) | The start time of R_1_(t) was uniformly randomly drawn from the observation period. The start time of R_3_(t) was uniformly randomly drawn from 21 to 56 days after the start time of R_1_(t). |  |
| V | RI(t) = R_1_(t - c_1_) + R_2_(t - c_2_) - 1 | The start time of R_1_(t) was uniformly randomly drawn from the observation period. The start time of R_2_(t) was uniformly randomly drawn from 21 to 56 days after the start time of R_1_(t). |  |
| VI | RI_total_(t) = R_2_(t - c_1_) + R_2_(t - c_2_) - 1  RI(t) = RI_total_(t)I(RI_total_ ≤ 4.5) + 4.5I(RI_total_ > 4.5) | The start time of the first risk period was uniformly randomly drawn from the observation period. The start time of the second risk period was uniformly randomly drawn from 14 to 28 days after the start time of R_1_(t). |  |

*Note*: ‘PLSI-SCCS’ denotes the partial linear single index SCCS method. ‘RI’ refers to relative incidence.

**Appendix 4:**

**Table S1: Simulation Results on Single Exposure Settings I and II Around the Peak.** Setting I: bell shaped $R_{1}(t)$. Setting II: PK/PD shaped $R_{2}(t)$.

| **Setting** | **N** | Method | **MISE** (SD) | **MAPE** (SD) |
| --- | --- | --- | --- | --- |
| I | 200 | PLSI-SCCS | 3.112 (1.694) | 0.902 (0.462) |
|  |  | Spline-based SCCS | 3.519 (1.655) | 1.017 (0.498) |
|  | 500 | PLSI-SCCS | 1.878 (0.977) | 0.546 (0.274) |
|  |  | Spline-based SCCS | 1.985 (1.260) | 0.626 (0.376) |
|  | 1000 | PLSI-SCCS | 1.524 (0.727) | 0.417 (0.203) |
|  |  | Spline-based SCCS | 1.443 (0.860) | 0.451 (0.251) |
| II | 200 | PLSI-SCCS | 3.221 (1.765) | 0.891 (0.474) |
|  |  | Spline-based SCCS | 3.007 (1.382) | 0.840 (0.387) |
|  | 500 | PLSI-SCCS | 1.952 (1.198) | 0.556 (0.329) |
|  |  | Spline-based SCCS | 2.759 (0.979) | 0.721 (0.266) |
|  | 1000 | PLSI-SCCS | 1.492 (0.699) | 0.389 (0.184) |
|  |  | Spline-based SCCS | 2.273 (0.789) | 0.590 (0.205) |

*Notes*: ‘MISE’: median integrated squared error. ‘MAPE’: mean absolute predicted error. ‘CP’: coverage probability. The peak for Setting I is defined as 15-25 days after the risk period starts. The peak for Setting II is defined as 5-15 days after the risk period starts.

**Table S2: Running time per replicate on Settings I and V.** Setting I: bell shaped $R_{1}(t)$. Setting V: Multiple exposure setting with overlapping risk periods; bell shaped $R_{1}(t)$ followed by PK/PD shaped $R_{2}(t)$ separated by uniform(21, 56) days. All models were fitted on a single CPU node of AMD EPYC 7763 64-Core Processor. The smoothness tuning parameter λ was fixed at a small value, since variations among small λ values had very little effect on running time. Bootstraps were not included.

| **Setting** | Method | M1 | M2 | N | **Time in seconds (SD)** |
| --- | --- | --- | --- | --- | --- |
| I | PLSI-SCCS | 8 | 4 | 200 | 24.83 (14.17) |
|  |  |  |  | 500 | 44.14 (27.37) |
|  |  |  | 7 | 200 | 60.18 (36.90) |
|  |  |  |  | 500 | 101.29 (47.31) |
|  |  | 10 | 4 | 200 | 37.26 (17.74) |
|  |  |  |  | 500 | 71.32 (37.89) |
|  |  |  | 7 | 200 | 81.61 (46.10) |
|  |  |  |  | 500 | 147.06 (72.57) |
|  | **Spline-based SCCS** | - | - | 200 | 1.99 (0.22) |
|  |  |  |  | 500 | 2.88 (0.21) |
| V | PLSI-SCCS | 8 | (4,4) | 200 | 173.71 (97.93) |
|  |  |  |  | 500 | 252.95 (146.65) |
|  |  |  | (8,8) | 200 | 378.78 (178.70) |
|  |  |  |  | 500 | 595.12 (285.70) |
|  |  | 10 | (4,4) | 200 | 196.69 (95.81) |
|  |  |  |  | 500 | 310.16 (155.34) |
|  |  |  | (8,8) | 200 | 339.35 (174.14) |
|  |  |  |  | 500 | 588.65 (244.27) |

*Notes*: For each simulation setting, 200 independent replicate datasets were generated. Reported times are the empirical mean and standard deviation across the 200 replicates.

#### Table S3: Estimates from the Standard SCCS Method on Hib, MMR and Febrile Convulsions Data.

| Variable | Estimate [95% CI] | Exponential Estimates [95% CI] |
| --- | --- | --- |
| Days after Hib vaccination |  |  |
| 0 to 5 | -0.46 [-1.35, 0.43] | 0.63 [0.26, 1.54] |
| 6 to 10 | 0.13 [-0.39, 0.64] | 1.13 [0.68, 1.91] |
| 11 to 15 | 0.11 [-0.53, 0.75] | 1.12 [0.59, 2.13] |
| 16 to 20 | -0.48 [-1.37, 0.40] | 0.62 [0.26, 1.49] |
| 21 to 25 | -0.29 [-1.10, 0.52] | 0.75 [0.33, 1.68] |
| 26 to 30 | -0.28 [-1.08, 0.53] | 0.76 [0.34, 1.70] |
| 31 to 35 | 0.59* [0.05, 1.12] | 1.80* [1.05, 3.07] |
| 36 to 40 | -0.10 [-0.85, 0.65] | 0.90 [0.43, 1.91] |
| 41 to 45 | 0.37 [-0.23, 0.98] | 1.45 [0.80, 2.65] |
| 45 to 49 | 0.19 [-0.48, 0.85] | 1.21 [0.62, 2.34] |
| Days after MMR vaccination |  |  |
| 0 to 2 | -0.63 [-1.38, 0.12] | 0.53 [0.25, 1.12] |
| 3 to 6 | 0.29 [-0.05, 0.63] | 1.33 [0.95, 1.87] |
| 7 to 10 | 1.20*** [0.98, 1.42] | 3.32*** [2.66, 4.14] |
| 11 to 15 | 0.30 [-0.00, 0.60] | 1.35 [1.00, 1.82] |
| 16 to 49 | 0.12 [-0.02, 0.26] | 1.12 [0.98, 1.29] |

*Note*: *: p < .05; **: p < .01; ***: p < .001.

**Figure S1: Predicted relative incidence curve for single exposure settings I and II.** Setting I: bell shaped $R_{1}(t)$. Setting II: PK/PD shaped $R_{2}(t)$.


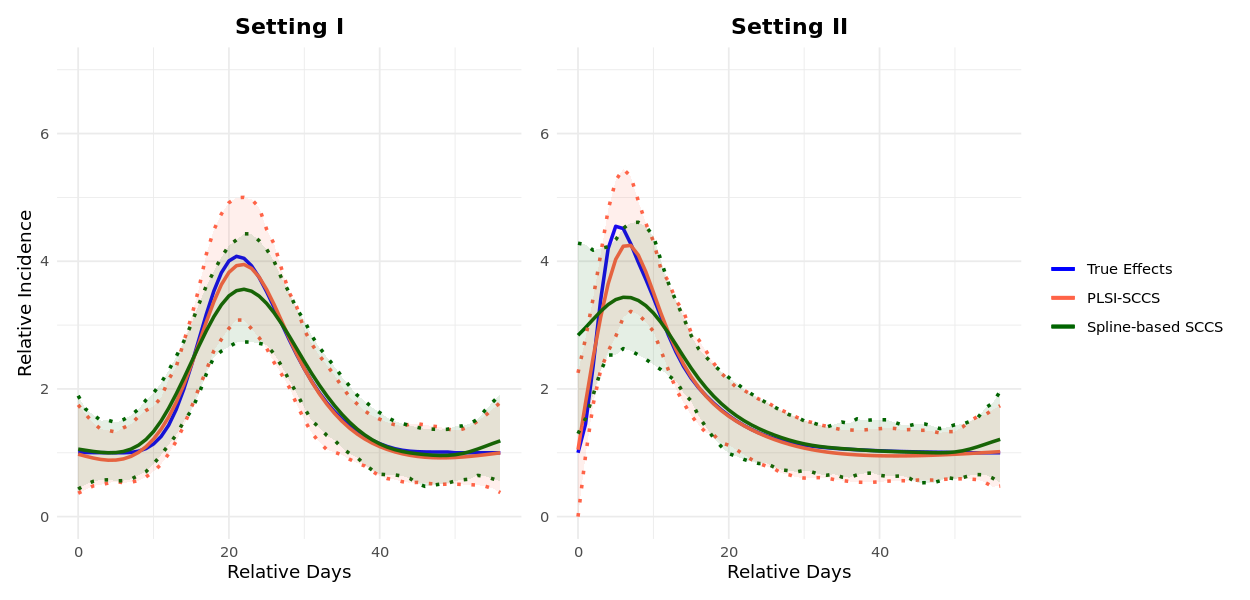


*Notes:* Shaded area covers 95% predictions across all replicates.

#### Figure S2: Predicted **relative incidence curve** for setting III. Setting III is the multiple exposure setting with non-overlapping risk periods. The first risk period is the bell shaped curve $\boldsymbol{R}_{\mathbf{1}}\mathbf{(}\boldsymbol{t}\mathbf{)}$. The latter is the PK/PD shaped curve $\boldsymbol{R}_{\mathbf{2}}\mathbf{(}\boldsymbol{t}\mathbf{)}$, starting at 56 days after the first risk period.

**
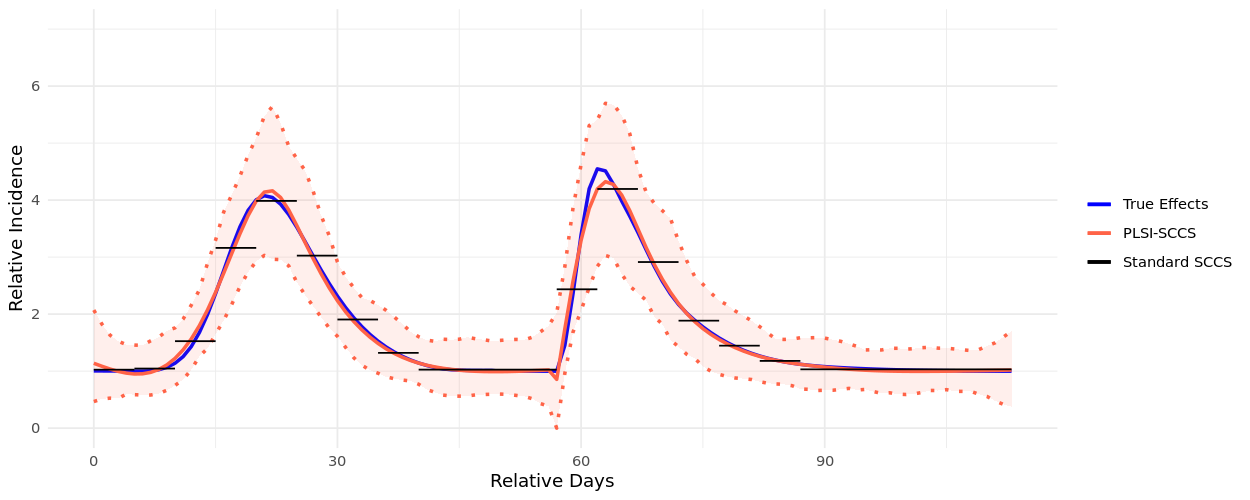
**

#### *Notes:* ‘Standard SCCS’ refers to the parametric self-controlled case series method where both exposure effects and age effects are approximated by step functions. Black lines denote model estimates from the standard parametric SCCS method. The Shaded area covers 95% predictions across all replicates. Details on how the step-function interval lengths were chosen for the standard SCCS are provided in Appendix 2.

#### Figure S3: Predicted relative incidence curves and exposure contributions in simulation setting IV. Setting IV is the multiple exposure setting with overlapping risk periods. The first risk period is the bell shaped curve $\boldsymbol{R}_{\mathbf{1}}\mathbf{(}\boldsymbol{t}\mathbf{)}$. The latter is the constant risk $\boldsymbol{R}_{\mathbf{3}}\mathbf{(}\boldsymbol{t}\mathbf{)}$. The start time of the second risk period was uniformly drawn from 21 to 56 days after the start time of the first.

####
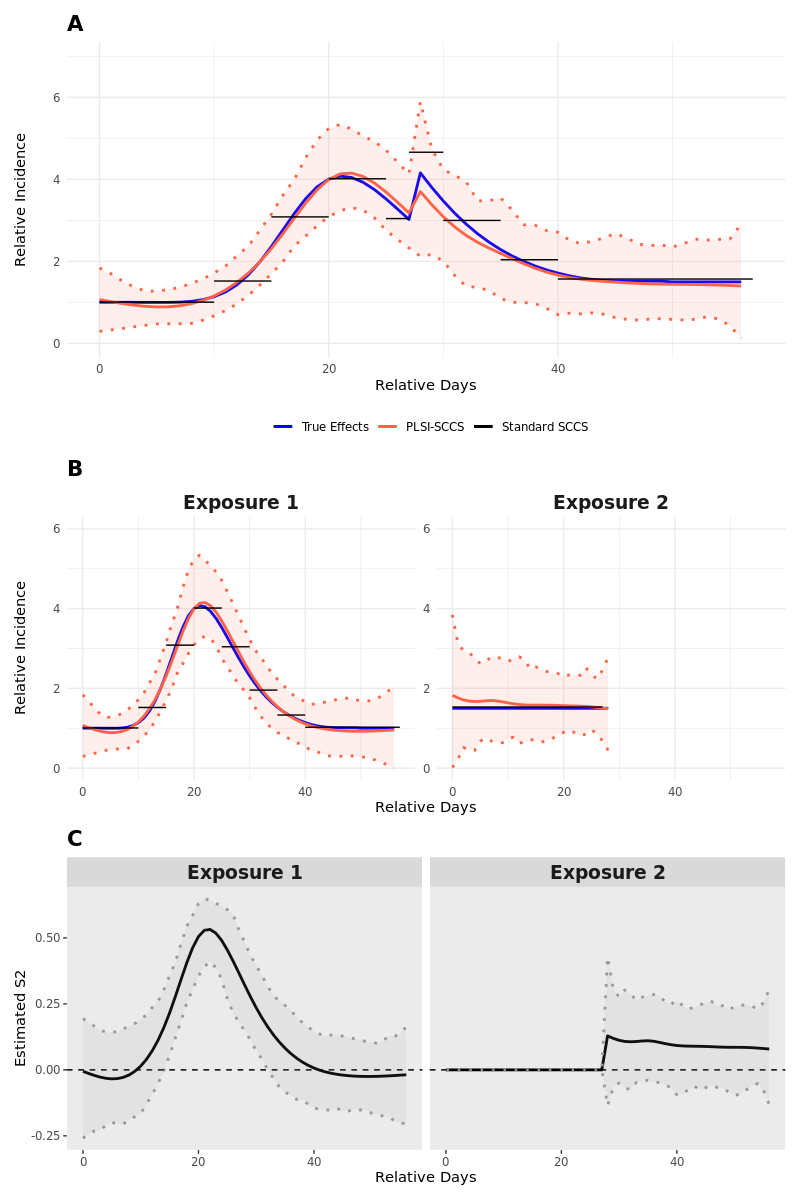


#### *Notes:* The shaded area covers the 95% prediction interval across 200 replicates. Panel A displays the overall prediction for exposure-related relative incidence (RI), assuming that the second risk period begins 28 days after the start of the first risk period. Panel B predicts exposure-related RI for each risk period. Panel C shows the estimated 2 curves for the relative importance of each exposure when the overlapping risk period begins 28 days after the first. For panel C, we multiply ${\hat{\text{S}}}_{\text{2}}$ of each replicate by the sign of its maximum absolute value to ensure that the first exposure is visualized as positive.

#### Figure S4: Distribution of Relative Days During the Risk Period in Data on Hib, MMR, and Febrile Convulsions. “MMR+Hib” denotes the number of events that occur during the risk period among the 212 children who receive both the MMR and Hib vaccinations on the same day.


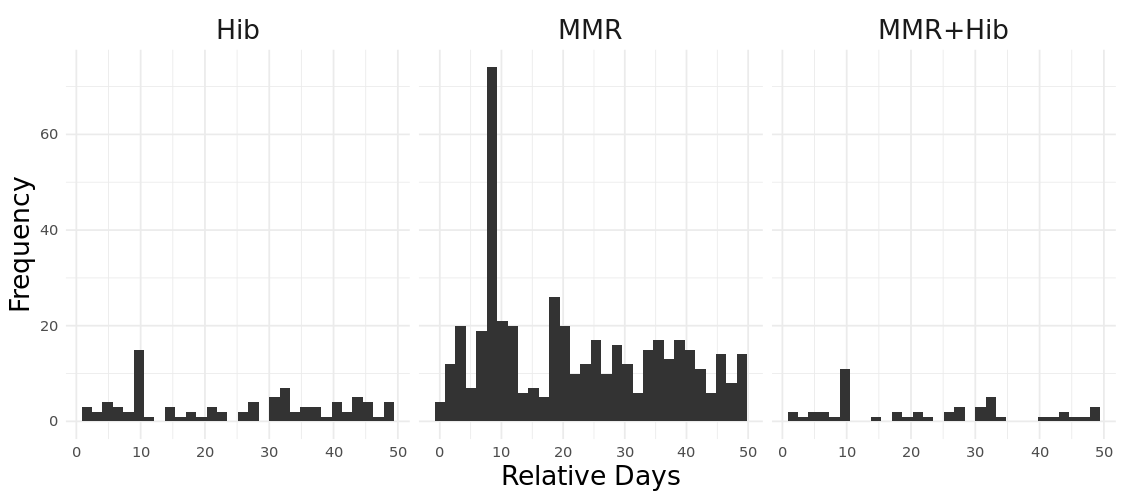


**Figure S5: Relationship between estimated** $\hat{S}_{2}$ **and predicted relative incidence in data analyses**. The left panel shows the linear and monotonically increasing relationship between observed $\hat{S}_{2}$ values and predicted relative incidence in the Hib, MMR vaccination, and febrile convulsions study. The right panel shows the monotonically decreasing relationship between observed $\hat{S}_{2}$ values and risk predictions in the malaria chemoprevention trial. Both indicate a monotonic $\hat{S}_{1}$ link function.

####
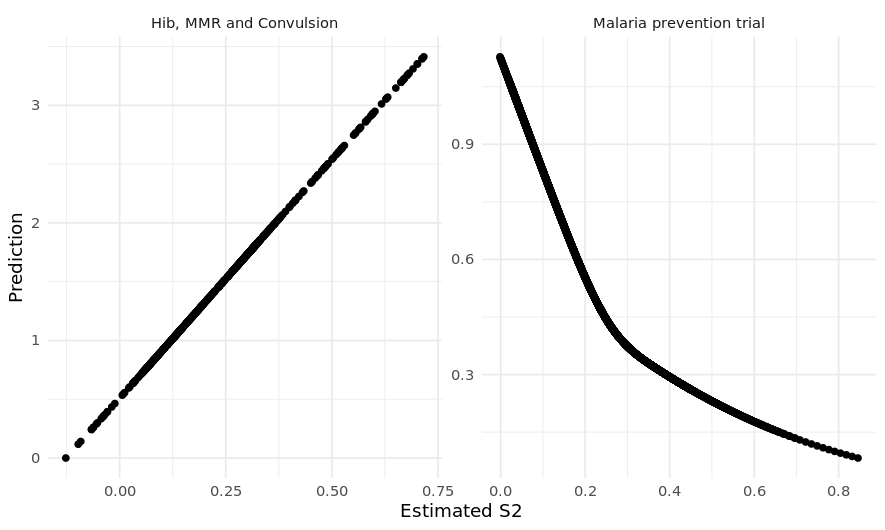

Supplement: Supplementary file 1 — Appendix S1: Supporting Information. [file SIM-45-0-s001.docx]
